# TNFAIP8 promotes the proliferation and cisplatin chemoresistance of non-small cell lung cancer through MDM2/p53 pathway

**DOI:** 10.1186/s12964-018-0254-x

**Published:** 2018-07-31

**Authors:** Ying Xing, Yuechao Liu, Tianbo Liu, Qingwei Meng, Hailing Lu, Wei Liu, Jing Hu, Chunhong Li, Mengru Cao, Shi Yan, Jian Huang, Ting Wang, Li Cai

**Affiliations:** 1 The Fourth Department of Medical Oncology, Harbin Medical University Cancer Hospital, 150 Haping Road, Harbin, 150040 China; 2 Department of Gynecology, Harbin Medical University Cancer Hospital, Harbin Medical University, 150 Haping Road, Harbin, 150040 China; 3 The Sixth Department of Medical Oncology, Harbin Medical University Cancer Hospital, 150 Haping Road, Harbin, 150040 China

**Keywords:** TNFAIP8, NSCLC, Proliferation, Cisplatin chemoresistance, MDM2/p53 pathway

## Abstract

**Background:**

The highly refractory nature of non-small cell lung cancer (NSCLC) to chemotherapeutic drugs is an important factor resulting in its poor prognosis. Recent studies have revealed that tumour necrosis factor alpha-induced protein 8 (TNFAIP8) is involved in various biological and pathological processes of cells, but their underlying mechanisms in processes ranging from cancer development to drug resistance have not been fully elucidated.

**Methods:**

TNFAIP8 expression in clinical NSCLC samples was examined through immunohistochemistry (IHC). After adjusting for patients’ characteristics with propensity score matching, Kaplan-Meier analysis and Cox regression analysis were performed for comparison of patients’ survival according to the TNFAIP8 level. Lentiviral transfection with TNFAIP8-specific shRNAs was used to establish stable TNFAIP8 knockdown (TNFAIP8 KD) NCI-H460, A549 and cis-diamminedichloroplatinum II resistant A549 (A549/cDDP) cell lines. Cell proliferation and viability were assessed by CCK-8 assay. Cell cycle was examined by flow cytometry. Multiple pathways regulated by TNFAIP8 KD were revealed by microarray analysis.

**Results:**

We found that high TNFAIP8 expression was associated with advanced pT stage, advanced pTNM stage, lymph node metastasis and unfavourable survival in NSCLC patients. TNFAIP8 shRNAs reduced in vitro cancer cell proliferation and in vivo tumor growth. Additionally, TNFAIP8 KD increased the sensitivity of NSCLC cells to cisplatin in vitro and in vivo. Conversely, up-regulation of TNFAIP8 promoted the proliferation and drug resistance to cisplatin of NSCLC cells. TNFAIP8 influences cancer progression pathways involving the MDM2/p53 pathway. Indeed, we observed that TNFAIP8 KD mediated the MDM2 downregulation and the p53 ubiquitination, thereby decreasing the degradation of p53 protein. shRNA p53 reversed TNFAIP8 shRNA-mediated regulation of cell proliferation, cell cycle, cisplatin sensitivity, and expression levels of RAD51, a DNA repair gene.

**Conclusion:**

Our work uncovers a hitherto unappreciated role of TNFAIP8 in NSCLC proliferation and cisplatin chemoresistance that is mediated through the MDM2/p53 pathway. These findings might offer potential therapeutic targets for reversing cisplatin resistance in NSCLC patients with high TNFAIP8 expression.

**Electronic supplementary material:**

The online version of this article (10.1186/s12964-018-0254-x) contains supplementary material, which is available to authorized users.

## Background

Lung cancer is the first leading cause of cancer-related mortality worldwide [1]. Non-small cell lung cancer (NSCLC) accounts for more than 70% of all lung cancers, and the five-year survival rate for patients with advanced stage IIIB or IV NSCLC is only 4% [2]. Cisplatin, a DNA-damaging cytotoxic agent, is the first-line therapy for treating advanced NSCLC, but chemoresistance has become a major obstacle to its use in clinical therapy [3, 4]. A better understanding of the molecular pathogenesis of NSCLC and the molecular mechanisms of cisplatin resistance will help to establish effective prognostic biomarkers and to improve the efficacy of related therapeutic interventions.

Tumour necrosis factor alpha-induced protein 8 (TNFAIP8), also called SCC-S2/GG2–1/NDED, acts as an anti-apoptotic and oncogenic molecule [5, 6]. TNFAIP8 overexpression in cancer cells is induced by TNF-α and NF-κB activation in various cells; these actions enhance cell survival and proliferation by inhibiting apoptotic protein caspase-8 and caspase-3 activity [7–9]. The available lines of evidence reveal TNFAIP8 overexpression in a variety of tumours, and this overexpression is associated with clinical parameters and experimental metastasis [10–15]. At present, studies of TNFAIP8 in NSCLC tumourigenesis and its clinical significance are lacking.

In a cohort of clinical samples, TNFAIP8 overexpression predicted platinum chemo- therapeutic responses in epithelial ovarian cancer [16]. TNFAIP8 was found to be overexpressed in patients with chemotherapy-resistant acute myeloid leukaemia [17]. A TNFAIP8 antisense oligonucleotide enhanced the antitumour efficacy of a combination of ionizing radiation or docetaxel in a prostate cancer xenograft model [6]. These findings suggest that TNFAIP8 might play a pivotal role in tumour chemotherapy resistance. However, the molecular mechanisms underlying the involvement of TNFAIP8 in chemotherapy resistance remain uncharacterized to date.

Oncoprotein murine double minute 2 (MDM2) promoted ubiquitination and proteasomal-dependent degradation of wild-type p53, which regulates cellular pathways such as DNA repair, cell cycle, apoptosis, angiogenesis, and senescence [18–20]. Lowe et al. [21] recently reported that the TNFAIP8 variant 2 regulates p53 by promoting its acetylation and localization to chromatin, but no studies have defined the relationship between TNFAIP8 and the p53 ubiquitination.

In this study, we analysed clinical NSCLC samples and determined that increased TNFAIP8 immunoreactivity in lung cancer patients was accompanied by decreased postoperative survival. We first performed gene expression profiling in lung cancer cells after shRNA knockdown of TNFAIP8 and analysed the gene expression data. The results revealed an association between TNFAIP8 expression and the MDM2/p53 pathway. TNFAIP8 induced cell proliferation in vitro and tumour growth in vivo by regulating MDM2, p53 and cyclin D1. We demonstrated that the knockdown of TNFAIP8 expression in NSCLC cells sensitized them to cisplatin by regulating the MDM2, p53 and RAD51 levels. TNFAIP8 thus represents a novel therapeutic target and biomarker for predicting treatment outcomes for NSCLC patients administered with cisplatin-based therapies.

## Methods

### Patients

From 2008 to 2009, we enrolled 196 NSCLC patients who underwent surgery in Harbin Medical University Cancer Hospital. 121 of them performed 3–4 cycles of platinum-based adjuvant chemotherapy after surgery. OS was calculated as the days from diagnosis to death and analyzed by the Kaplan-Meier method with log-rank test. 20 pairs of fresh lung tissues (paired NSCLC tumor samples and matched adjacent normal tissue samples) were resected from 20 NSCLC patients who underwent surgical lung resection between June 2015 and June 2016. Normal lung tissue samples were taken from areas a standard distance (3 cm) from resected NSCLC patients [22]. Our study was approved by the Institutional Review Board of the Harbin Medical University Cancer Hospital. All patients provided informed consent prior to the study.

### Immunohistochemistry

Paraffin-fixed tissue sections were stained for TNFAIP8 immunohistochemistry using anti-TNFAIP8 antibody (ab64988, Abcam, Cambridge, MA, USA) and at a dilution of 1:100 overnight at 4 °C. The next day after washing with PBS, the sections subjected to a streptavidin-biotin complex system. For all stains, sections were counterstained in Mayer’s haematoxylin for 1 min, dehydrated, cleared and coverslipped using mounting media. The staining results were scored based on previously described criteria [12].

### Cell culture

The human NSCLC cell lines A549, NCI-H1666, NCI-H2170, NCI-H520, NCI-H1650, NCI-H1792, NCI-H1975, HCC827 and NCI-H460 were obtained from the American Type Culture Collection (ATCC, Manassas, VA, USA) and propagated in RPMI 1640 medium (HyClone, Logan, UT, USA) at 37 °C in a humidified atmosphere of 5% CO_2_. Stable cisplatin-resistant A549 cells (A549/cDDP) were established by continuous culture for 8 months in the selection of cisplatin (Sigma, St. Louis, MO, USA) from 0.3 to 20 μg/mL as previously described [23]. According to the manufacturer’s instructions, stable cells expressing shRNA of TNFAIP8 or p53 were obtained by transducing cells with lentiviral expression vectors and culturing in the 1640 medium containing 1 μg/mL puromycin (Sigma, St. Louis, MO, USA).

### qRT-PCR

Immediately following resection, fresh tissues were stored at − 80 °C until RNA extraction. Total RNA from fresh frozen samples or lung cells was extracted using RNAeasy kit (Qiagen, Valencia, CA, USA). The RNA quantification was measured using a NanoDrop 2000 spectrophotometer (Thermo, Wilmington, DE, USA). Reverse transcription was performed using the High Capacity Reverse Transcription kit (PR1702, BioTeke, Beijing, China) according to the manufacturer’s instructions. DNA was quantified using Fast SYBR Green Master Mix (Applied Biosystems). The specific sequences of the primers used were as follows: 5’-TGAAGATGGAGCACTGCTGA-3′ (forward) and 5’-GGTCTGTTACCCGTTAGGAAG-3′ (reverse) for TNFAIP8; 5’-ATGGAACATCAGCTGCTGT-3′ (forward) and 5’-TCAGATGTCCACATCCCGC-3′ (reverse) for cyclin D1; 5’-GGCGCCTATGGGAAGGTGTTC-3′ (forward) and 5′- AAAGTCCAGACCTCGGAGAAGC-3′ (reverse) for CDK6; 5’-GCTGCGGACCGAGTAATG-3′ (forward) and 5′- CCAGCTTCTTCCAATTTCTTCAC-3′ (reverse) for RAD51; 5’-TGGCGTGCCAAGCTTCTCTGT-3′ (forward) and 5’-ACCTGAGTCCGATGATTCCTGCT-3′ (reverse) for MDM2; 5’-GCAACTATGGCTTCCACCTG-3′ (forward) and 5′- CAGAGAGCACCGCGACCACG-3′ (reverse) for p53. β-actin was applied as housekeeping gene, its primers were as follows: forward, 5’-CTTAGTTGCGTTACACCCTTTCTTG-3′; reverse, 5’-CTGTCACCTTCACCGTTCCAGTTT-3′. Quantitative normalization of target cDNA was perormed for each sample using β-actin expression as an internal control. The relative levels of TNFAIP8, cyclin D1, CDK6, RAD51, MDM2, p53 vs. β-actin were determined by the comparative CT (the 2^−ΔΔ CT^) method.

### Western blot analysis

Total proteins from frozen tissues or cells were extracted using RIPA buffer containing a 1% protease inhibitors (Roche). Protein concentration was determined by Bradford assay (Thermo Scientific, Waltham, MA, USA). Equal amount of proteins were separated in 12% SDS-polyacrylamide gels and transferred onto PVDF membranes. Next, we blocked the membrane with 5% non-fat dry milk in Tris-buffered saline and Tween 20 (10 mM Tris-HCl, pH 8.0, 100 mM NaCl and 0.05% Tween, TBS-T), and incubated the membrane at 4 °C overnight with primary antibodies, followed by with horseradish peroxidase (HRP)-conjugated secondary antibodies. The immunoreactivities were visualized by enhanced chemiluminescence (ECL) kit according to manufacturer’s instruction. Western blot analyses were performed using primary antibodies against TNFAIP8 (ab166804, Abcam, Cambridge, MA, USA), MDM2 (ab38618, Abcam, Cambridge, MA, USA), p53 (ab179477, Abcam, Cambridge, MA, USA), cyclin D1 (ab134175, Abcam, Cambridge, MA, USA), CDK6 (ab124821, Abcam, Cambridge, MA, USA) and RAD51 (ab133534, Abcam, Cambridge, MA, USA). Western blot bands were quantified by the ImageJ software (U.S. National Institutes of Health, USA). The experiment was repeated thrice.

### Microarray processing and analysis

Total RNA from NCI-H460 cells infected with lentivirus expressing either Ctrl-shRNA or TNFAIP8-shRNA2 was extracted using Trizol reagent. Affymetrix GeneChip® PrimeView™ Human Gene Expression Microarray analysis was used, according to manufacturer’s instruction. Pathway enrichment analysis was conducted for differentially expressed genes using IPA commercially available software.

### Immunofluorescence

The cells were fixed with 4% paraformaldehyde for 20 min, permeabilized with 0.3% Triton X-100 for 10 min, and blocked by 1% normal goat serum for 2 h at room temperature. Then, the cells were incubated with RAD51 antibodies (1:100 dilution; ab133534, Abcam, Cambridge, MA, USA) overnight at 4 °C. Next day, the cells were incubated with Alexa Fluor 488-labelled goat anti-rabbit secondary antibody (ab150077, Abcam, Cambridge, MA, USA) for 1 h and staining with DAPI (Beyotime, Shanghai, China) for 5 min at room temperature. During each step, cells were washed three times with PBS. In antifade mounting medium the stained cells were mounted, and then pictures were taken under a fluorescence microscope.

### Immunoprecipitation (IP) assay

IP were performed with Crosslink Magnetic IP/Co-IP Kit (Thermo, Rockford, IL, USA). According to the manufacturer’s instructions, prewash beads two times with 1X Modified Coupling Buffer and bind 5 μ g of a relevant primary antibody to beads for 15 min. Next, wash beads three times with 1X Modified Coupling Buffer and crosslink antibody to beads with DSS for 30 min. Then, wash beads 3 times with Elution Buffer followed by two washes with IP Lysis/Wash Buffer. Incubate cell lysate with antibody-crosslinked beads for 1–2 h at room temperature or overnight at 4 °C. At last, wash beads two times with IP Lysis/Wash Buffer and one time with ultrapure water, elute bound antigen, and subjected to western blot analysis.

### Ubiquitination assay

Cells pretreated with 25 μM MG132 for 6 h were lysed and incubated with anti-p53 antibodies overnight at 4 °C. The immunoprecipitates were dissolved in 30 μl 5 × SDS protein loading buffer and analysed by western blot analysis using an anti-ubiquitin antibody (1:100 dilution; ab134953, Abcam, Cambridge, MA, USA).

### Cell viability assay

The viability of cells was measured using the Cell Counting Kit-8 (CCK-8) (Dojindo Molecular Technologies, Kumamoto, Japan). At a density of 7 × 10^3^ cells per well, the cells were cultured in a 96-well plate. After 24, 48, 72, or 96 h of growth the cells were incubated in 10% CCK-8 solution for an additional 1 h at 37 °C in dark; or at the same condition, after cisplatin treatment, 10 μL of CCK-8 reagent was added to each well. The absorbance at 450 nm (A450) was examined on a microplate reader (BioTek, Winooski, VT, USA). Three parallel experiments were performed in 6 replicate wells per sample. The IC50 values were determined using IBM SPSS Statistics 19.0 software.

### Flow cytometry and cell cycle analysis

Cells were trypsinized into single cell suspensions and fixed with 70% ethanol for 2 h. Then, cells were stained with FxCycle PI/RNase staining solution (BD Pharmingen, San Diego, CA, USA) and analysed using an LSR II flow cytometer (BD). Using FlowJo software (FlowJo LLC, Bethesda, USA), cell cycle analysis and model fitting were performed.

### Xenograft models

All experimental procedures and postoperative animal care were conducted in accordance with the Care and Use of Laboratory Animals (National Institutes of Health, revised 1985). A549/Ctrl or A549/TNFAIP8-sh2 cells (5 × 10^6^ per mice) were prepared in 0.2 mL of PBS and injected into the flanks of BALB/c nude mice under isoflurane anaesthesia (Nu/Nu, female, 4–6 weeks old, *n* = 10/group). Seven days later, the mice were treated with PBS or cDDP (3.0 mg/kg body weight; i.p., three times per week). By measuring the perpendicular tumour diameter (length (L) and width (W)) with Vernier callipers, tumour volume was measured every 3 days for 21 days. The tumour volume (V) was calculated using the following formula: V = LW^2^/2. At the last day, the tumour tissues were removed. Part of the tumour tissues were frozen in liquid nitrogen and stored at − 80 °C; the remaining were fixed in 4% paraformaldehyde and stored in 70% ethanol. The protocol was approved by the Institutional Ethics Committee for the Administration of Laboratory Animals of Harbin Medical University, China.

### Statistical analysis

All statistical analyses were performed with SPSS 22.0 (SPSS, Chicago, IL, USA). The means of normally distributed continuous data between two groups were analysed by Student’s t-tests. The differences in categorical variables among different groups were analysed with χ^2^ tests. Survival curves were plotted using Kaplan-Meier method and compared by log-rank tests. The significant covariates found in univariate analysis were subjected to further multivariate analysis. Multivariate analysis of independent prognostic factors for OS and DFS were performed using Cox proportional hazards model. A two-tailed *P* value less than 0.05 was considered statistically significant.

## Results

### TNFAIP8 expression level in NSCLC tissues

**Fig. 1:**
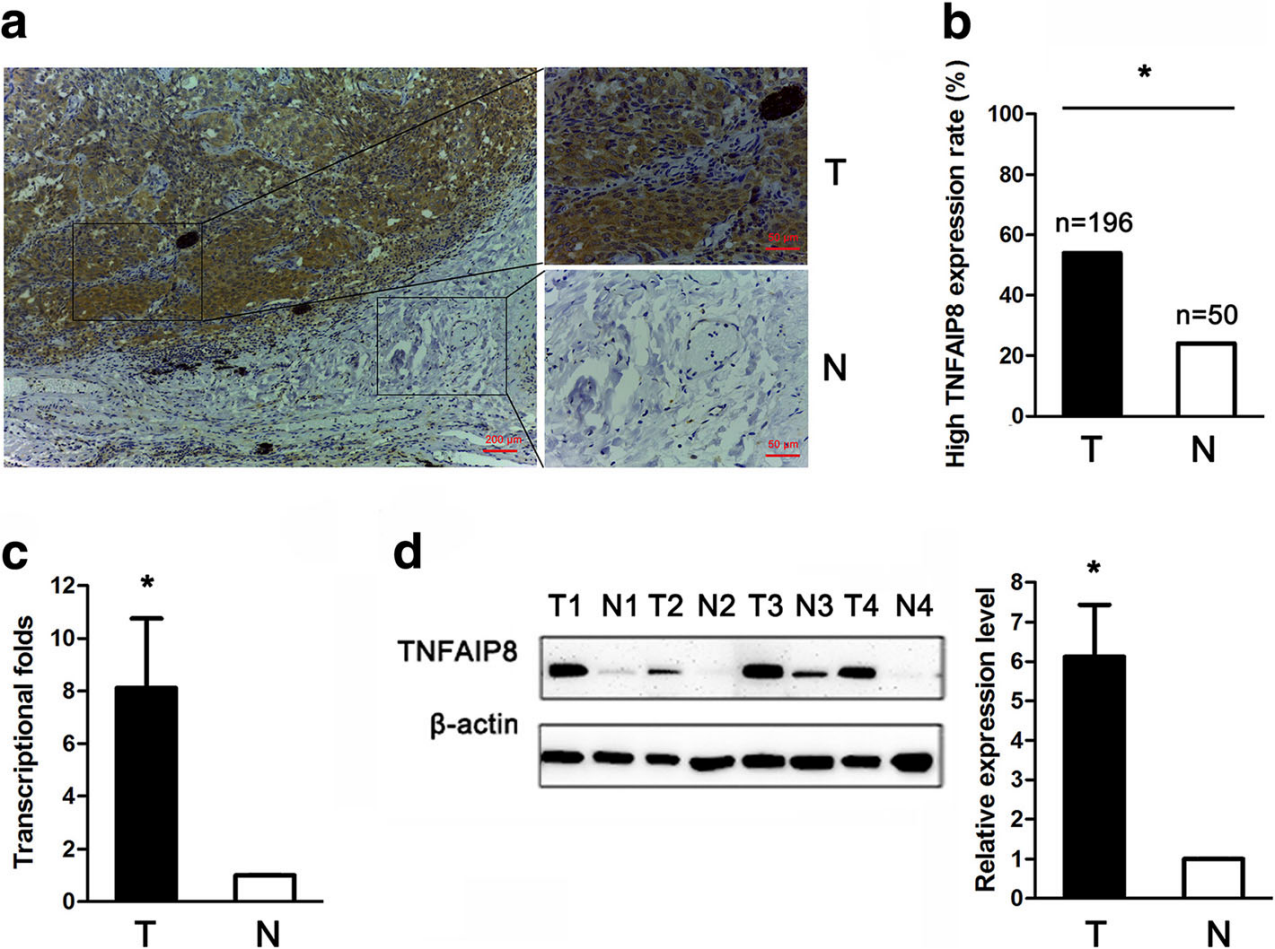
Tumor necrosis factor-a-induced protein 8 elevated expression in clinical NSCLC tissues. **a** Representative IHC images from a single NSCLC case (T) and the matched adjacent normal lung tissue (N). The protein expression levels of TNFAIP8 were significantly higher in tumour tissues than that in adjacent normal lung tissues. **b** Histogram showing pooled data derived from NSCLC (T, *n* = 196) and normal lung (N, *n* = 50) tissues. High TNFAIP8 expression rate is calculated by dividing the number of patients with high TNFAIP8 expression by the number of all patients. *P* values were calculated using the χ^2^ test. **c** Histogram showing TNFAIP8 mRNA expression in NSCLC (T, *n* = 20) tissues and adjacent normal lung tissue (N, n = 20). Normalization: The TNFAIP8/actin ratio was first calculated and set as 1.00. **d** Representative western blot showing TNFAIP8 expression in lung tissues and a histogram showing pooled data from NSCLC (T, n = 20) tissues and adjacent normal lung tissues (N, n = 20). Data are expressed as the mean ± SEM (*n* = 3). *P* values were calculated using Student’s t-test

TNFAIP8 was mainly localized to the cytoplasmic compartment of tumour cells (Additional file 1: Figure S1). TNFAIP8 was high expression in 54.1% of all NSCLC patients (106/196). The TNFAIP8 protein expression levels were significantly increased in tumour tissues compared with adjacent normal lung tissues (54.1% vs. 24.0%, respectively; Fig. 1a, b). Next, we examined TNFAIP8 expression in fresh tumour and normal tissues by quantitative real-time reverse transcription-polymerase chain reaction (qRT-PCR) and found that the mean relative TNFAIP8 mRNA expression levels were significantly increased in tumour tissues (*n* = 20) compared with normal lung tissues (n = 20); indeed, tumour tissues exhibited ~ 8.1-fold increased TNFAIP8 mRNA levels compared with normal tissues (Fig. 1c). TNFAIP8 protein expression in tumour and normal tissues was then examined through Western blot analyses. These results revealed higher protein expression levels of TNFAIP8 in NSCLC tissues compared with normal lung tissues (Fig. 1d).

### TNFAIP8 expression is an unfavourable predictor for survival

IHC analyses revealed that increased TNFAIP8 expression was correlated with advanced pT classification, advanced pTNM stage and the presence of positive lymph nodes (Table 1).

**Table 1:** Association between TNFAIP8 expression and clinicopathological characteristics of NSCLC patients

Variable	All patients	TNFAIP8 expression		*P*
		High (%)	Low (%)	
	(*n* = 196)	(*n* = 106)	(*n* = 90)	
Smoking				
Never	93	53 (50)	40 (44)	0.438
Ever	103	53 (50)	50 (56)	
Gender				
Male	129	68 (64)	61 (68)	0.594
Female	67	38 (36)	29 (32)	
Age (years)				
< 60	129	71 (67)	58 (64)	0.709
≥ 60	67	35 (33)	32 (36)	
Differentiation				
Well	18	12 (11)	6 (7)	0.263
Moderate	87	42 (40)	45 (50)	
Poor	91	52 (49)	39 (43)	
Histological cell type				
Adenocarcinoma	122	70 (66)	52 (58)	0.235
Squamous cell carcinoma	74	36 (34)	38 (42)	
pStage				
I	93	43 (40.5)	50 (56)	0.022*
II	40	20 (19)	20 (22)	
III	63	43 (40.5)	20 (22)	
pT classification				
T1	51	20 (19)	31 (34)	0.034*
T2	129	75 (71)	54 (60)	
T3/4	16	11 (10)	5 (6)	
Lymph node metastasis				0.005*
Present	82	54 (51)	28 (31)	
Absent	114	52 (49)	62 (69)	
Adjuvant therapy				
Yes	121	62 (58)	59 (66)	0.311
No	75	44 (42)	31 (34)	

*Abbreviations*: *NSCLC* non-small cell lung cancer, *pTNM stage* tumor, node, metastasis (pathological stage), *pT* pathological T stage, *n* number of patients. Ever: smoking at any time from the beginning of life. *P* value: the difference of clinicopathological characteristics between the TNFAIP8 high expression group and low expression group. *P < 0.05 was considered statistically significant

**Fig. 2:**
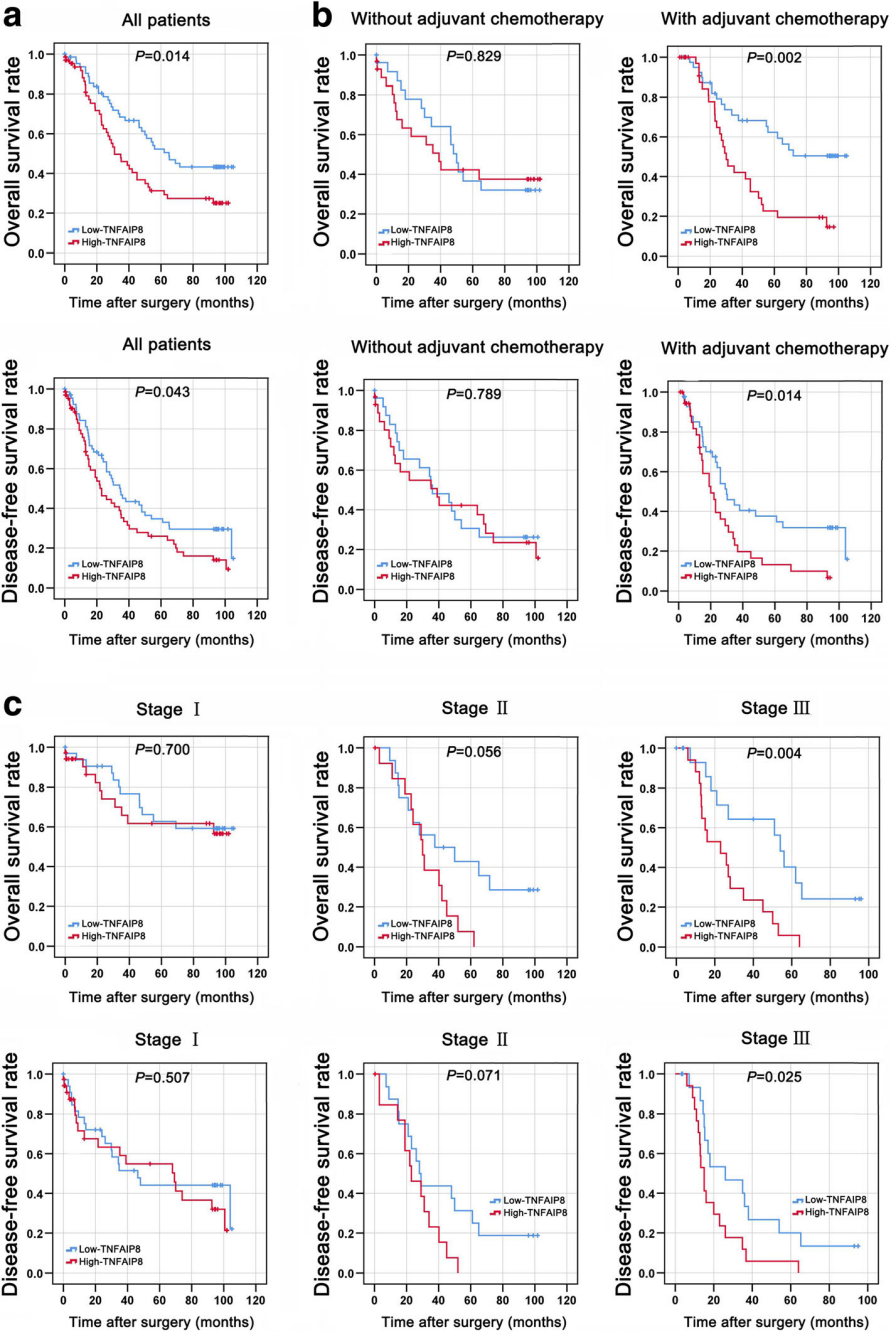
TNFAIP8 expression predicted survival in NSCLC. **a** High TNFAIP8 levels are associated with shorter survival in patients with NSCLC. Kaplan-Meier curves showing OS (top) or DFS (bottom) for patients with high and low levels of TNFAIP8 expression. **b** Kaplan-Meier plots of OS (top) or DFS (bottom) based on the expression levels of TNFAIP8 in NSCLC patients with or without adjuvant chemotherapy. **c** Survival curves showing the correlation of TNFAIP8 levels with OS (top) or DFS (bottom) in NSCLC patients in different pStage (Stage I, Stage II and Stage III)

To determine whether TNFAIP8 expression is a prognostic factor for overall survival (OS) and disease-free survival (DFS) in NSCLC, we performed Kaplan-Meier analyses and found that high TNFAIP8 expression predicts a poor prognosis in terms of both OS (Fig. 2a, top) and DFS (Fig. 2a, bottom). The patients were also divided into an adjuvant chemotherapy-treated group (*n* = 121) and a non-adjuvant chemotherapy-treated group (*n* = 75) based on whether they received adjuvant chemotherapy immediately after surgical resection. The OS yielded a significantly better prognosis for the adjuvant chemotherapy-treated patients with low TNFAIP8 expression compared with those with high TNFAIP8 expression (Fig. 2b). Using a stage-stratified analysis, we found that high TNFAIP8 expression might be an unfavourable predictor for Stage III NSCLC (Fig. 2c).

We subsequently performed univariate and multivariate analyses (Additional file 2: Table S1). The univariate analysis results revealed that advanced pTNM stage, positive lymph nodes and higher TNFAIP8 expression were indicators of poor OS; in addition, the adenocarcinoma subtype, advanced pTNM stage, positive lymph nodes and high TNFAIP8 expression were predictors for poor DFS. The multivariate analysis results demonstrated that high TNFAIP8 expression (HR, 1.858; 95% CI, 1.164–2.966; *P* = 0.009) was independent predictor for OS. High TNFAIP8 expression (HR, 1.605; 95% CI, 1.056–2.440; *P* = 0.027) was an independent indicator of DFS.

### TNFAIP8 promotes NSCLC cell proliferation and cell cycle progression

**Fig. 3:**
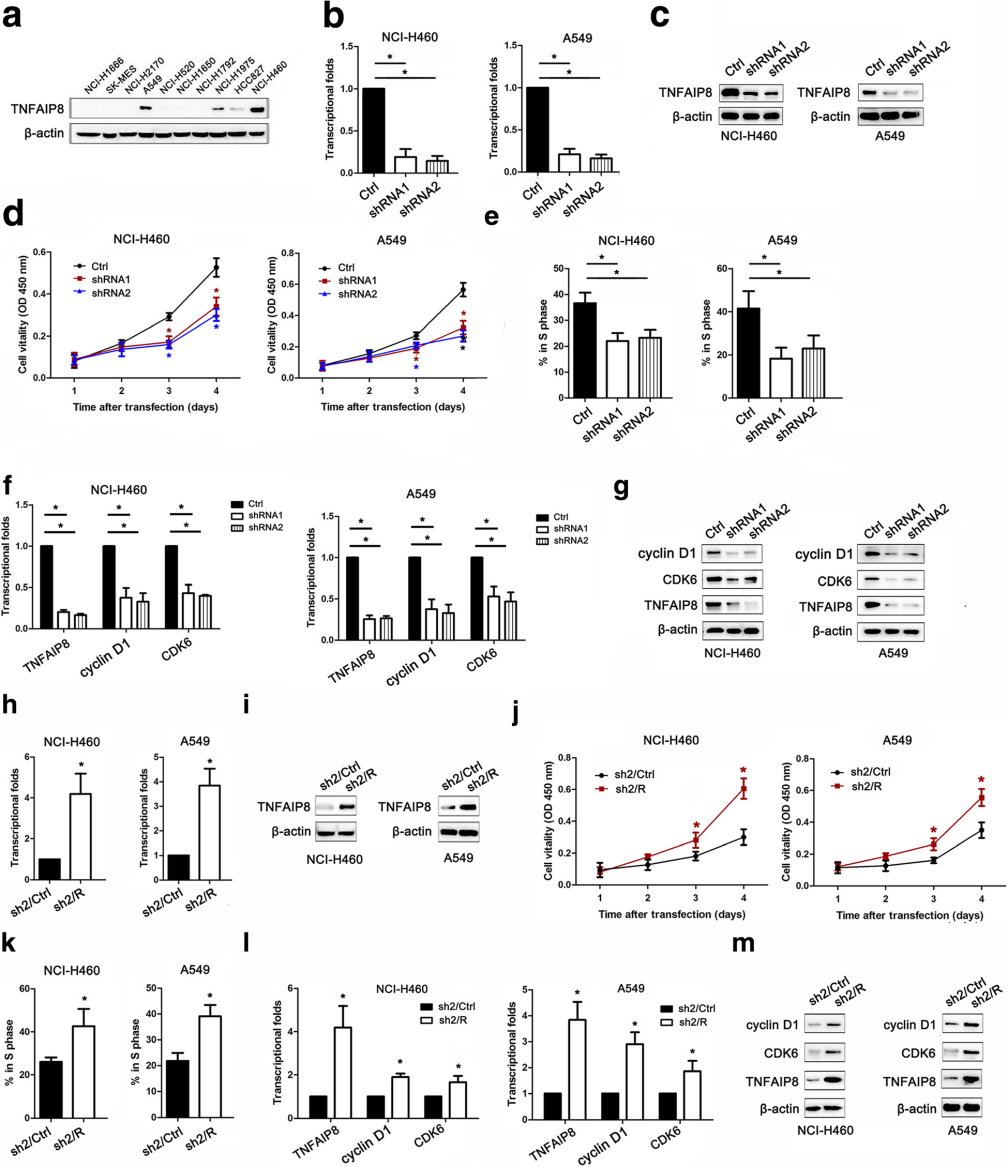
TNFAIP8 knockdown inhibits NSCLC cell proliferation and cell cycle progression. **a** TNFAIP8 expression levels in 10 lung cancer cell lines were examined by western blotting. β-actin was used as a loading control. **b, c** qRT-PCR and immunoblot analyses of TNFAIP8 mRNA and protein expression levels, respectively, in NCI-H460 (left panel) and A549 (right panel) cells and in cells transfected with control shRNA (Ctrl) or TNFAIP8 shRNAs (shRNAs). *P < 0.05 (Student’s t-test). **d** CCK-8 assay was used to examine changes in the proliferation rate of NSCLC cells at different time intervals (from 24 to 96 h). Data are expressed as the absorbance (mean ± SEM) for each group (n = 3). *P < 0.05 (Student’s t-test). **e** Cell cycle profiles were determined using FACS analysis. The mean percentage of cells in the S phase is shown. Error bars represent the standard error of the mean. *P < 0.05 (Student’s t-test). **f, g** qRT-PCR and representative western blot showing the effects of TNFAIP8 knockdown on the expression levels of cyclin D1 and CDK6 in NSCLC cells. **h, i** Real-time qRT-PCR and immunoblot analyses of TNFAIP8 mRNA and protein expression levels, respectively, we transiently transfected TNFAIP8 KD cells with the empty vector as control (designated sh2/Ctrl), or a vector encoding for human TNFAIP8 to restore TNFAIP8 expression (designated sh2/R) in NCI-H460 (left panel) and A549 (right panel) cells. *P < 0.05 (Student’s t-test). **j** CCK-8 assay was used to examine changes in the proliferation rate of NSCLC cells at different time intervals (from 24 to 96 h). Data are expressed as the absorbance (mean ± SEM) for each group (*n* = 3). *P < 0.05 (Student’s t-test). **k** Cell cycle profiles were determined using FACS analysis. The mean percentage of cells in the S phase is shown. Error bars represent the standard error of the mean. *P < 0.05 (Student’s t-test). **l, m** qRT-PCR and western blot analysis of TNFAIP8 and cyclin D1 and CDK6 in sh2/Ctrl or sh2/R cells. β-actin is shown as a loading control. All experiments were performed at least three times, and all samples were assayed in triplicate

Western blot analyses were performed to measure the TNFAIP8 protein levels in the lung cancer cell lines examined (Fig. 3a). NCI-H460 and A549 cells were selected as “loss-of-function” models because they expressed high levels of TNFAIP8. As shown in Fig. 3b, the knockdown of TNFAIP8 mRNA and protein by lentiviral transfection with TNFAIP8-specific shRNAs were more efficient than lentiviral transfection with control shRNA (Fig. 3b, c). IHC analysis confirmed that increased TNFAIP8 expression was significantly associated with pT classification. Therefore, we then used CCK-8 assay to test the effect of TNFAIP8 on NSCLC cell proliferation. TNFAIP8 KD significantly inhibited the proliferation of NCI-H460 and A549 cells compared with control cells (Fig. 3d). A cell cycle distribution analysis revealed that TNFAIP8 silencing reduced the percentage of cells in the S phase (Fig. 3e). We subsequently examined the expression levels of cell cycle-related proteins after treatment with TNFAIP8 shRNAs or control shRNA. Positive correlations between TNFAIP8 expression and cyclin D1 and CDK6 expression were noted, indicating that TNFAIP8 plays a key role in the regulation of the G1/S transition (Fig. 3f, g).

To validate the effect of TNFAIP8 overexpression on the NSCLC cell proliferation, we conducted a rescued expression experiment in which TNFAIP8 KD NSCLC cells were transiently transfected with a vector encoding for the human TNFAIP8 gene (designated sh2/R), or transfected with the empty vector as control (designated sh2/Ctrl) (Fig. 3h, i). Restoration of TNFAIP8 expression in TNFAIP8 KD NSCLC cells resulted in the increased cell proliferation (Fig. 3j). Moreover, TNFAIP8 overexpression accelerated entry into S phase (Fig. 3k). The reappearances of the levels of cyclin D1 and CDK6 to the levels of the control cells were observed in TNFAIP8-rescued cells (Fig. 3i-m).

### Effect of TNFAIP8 on cisplatin sensitivity

**Fig. 4:**
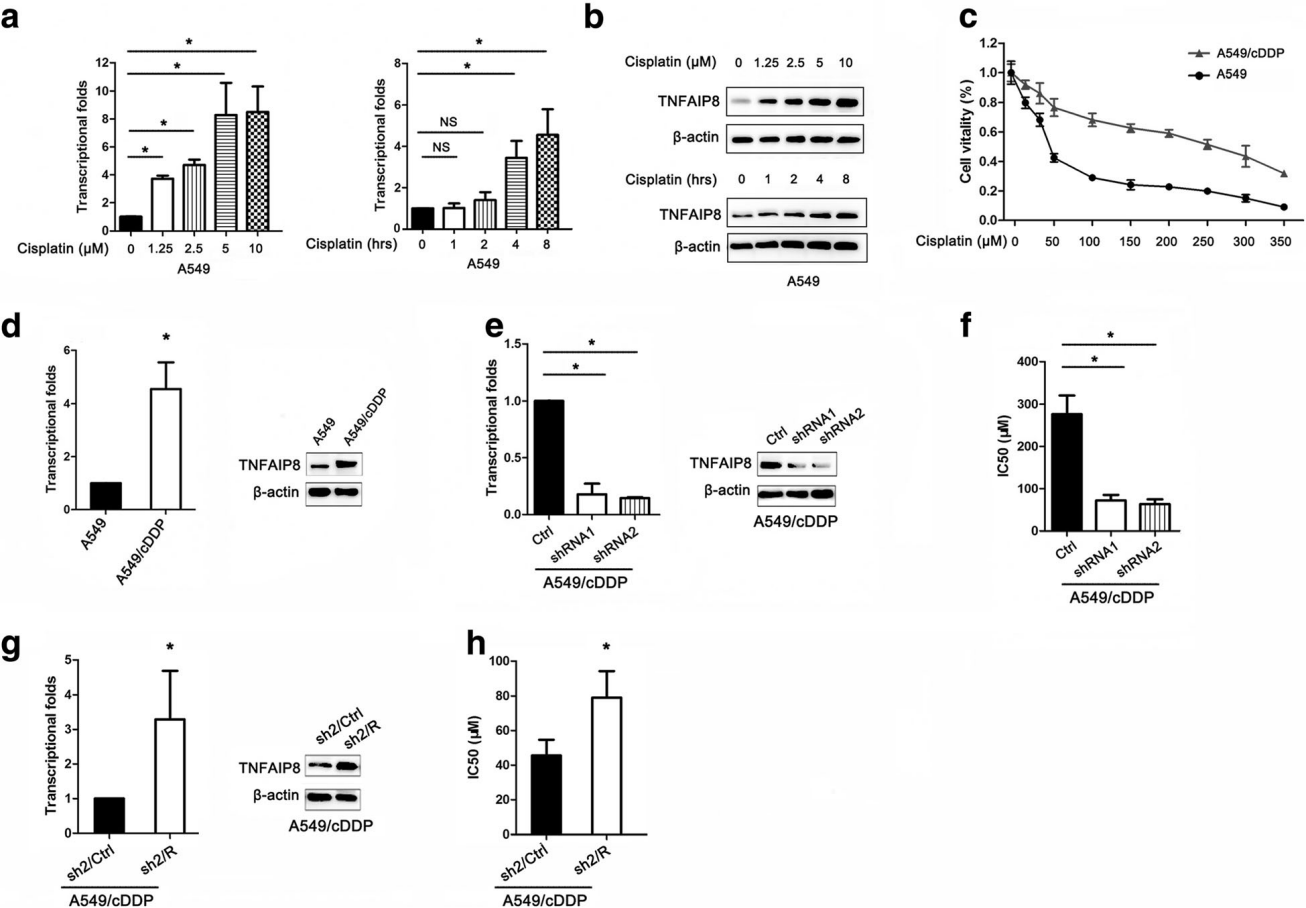
TNFAIP8 knockdown enhances the chemosensitivity of NSCLC cells to cisplatin. **a**, **b** Cisplatin induced TNFAIP8 expression in a time- and dose-dependent manner. **c** Parental A549 and the cDDP-resistant cell line (A549/cDDP) cells were treated with the indicated doses of cisplatin. **d** TNFAIP8 expression was confirmed at both the transcript and protein levels in the A549 and A549/cDDP cell lines. **e** A549/cDDP cells transfected with control shRNA (Ctrl) or TNFAIP8 shRNAs (shRNAs) was analysed for TNFAIP8 expression by qRT-PCR (left panel) and immunoblot assay (right panel). **f** CCK-8 assay results showed that TNFAIP8 knockdown in both A549 and A549/cDDP cells significantly decreased the IC50. Data are expressed as the mean ± SEM (n = 3). *P < 0.05 (Student’s t-test). **g** TNFAIP8 KD cells transiently transfected with the empty vector as control (designated sh2/Ctrl), or a vector encoding for human TNFAIP8 to restore TNFAIP8 expression (designated sh2/R) in A549/cDDP cells. *P < 0.05 (Student’s t-test). **h** IC 50 of cisplatin in sh2/Ctrl or sh2/R. The IC 50 values were obtained by CCK-8 assay. Data are expressed as the mean ± SEM (*n* = 3). *P < 0.05 (Student’s t-test)

Given that low TNFAIP8 expression was associated with a better response to chemotherapy in our patient cohort, we focused on its role in the chemosensitivity of NSCLC cells to cisplatin. Interestingly, cisplatin treatment substantially increased the TNFAIP8 mRNA and protein levels in A549 cells in a dose- and time-dependent manner (Fig. 4a, b). Moreover, TNFAIP8 expression was significantly increased in the A549/cDDP cells, cisplatin-resistant subclone, compared with the cisplatin-sensitive cell line (A549) (Fig. 4c, d). The above findings indicate that TNFAIP8 is correlated with the chemoresistance of NSCLC cells to cisplatin. After transfection with TNFAIP8 shRNAs, the IC50 values of A549 and A549/cDDP cells to cisplatin were both significantly decreased, as evidenced by the CCK8 assay results (Fig. 4e, f). Conversely, the resistance to cisplatin of the TNFAIP8 KD A549/cDDP cells was rescued by TNFAIP8 overexpressing (Fig. 4g, h).

### Identification of enriched pathways after TNFAIP8 KD

**Fig. 5:**
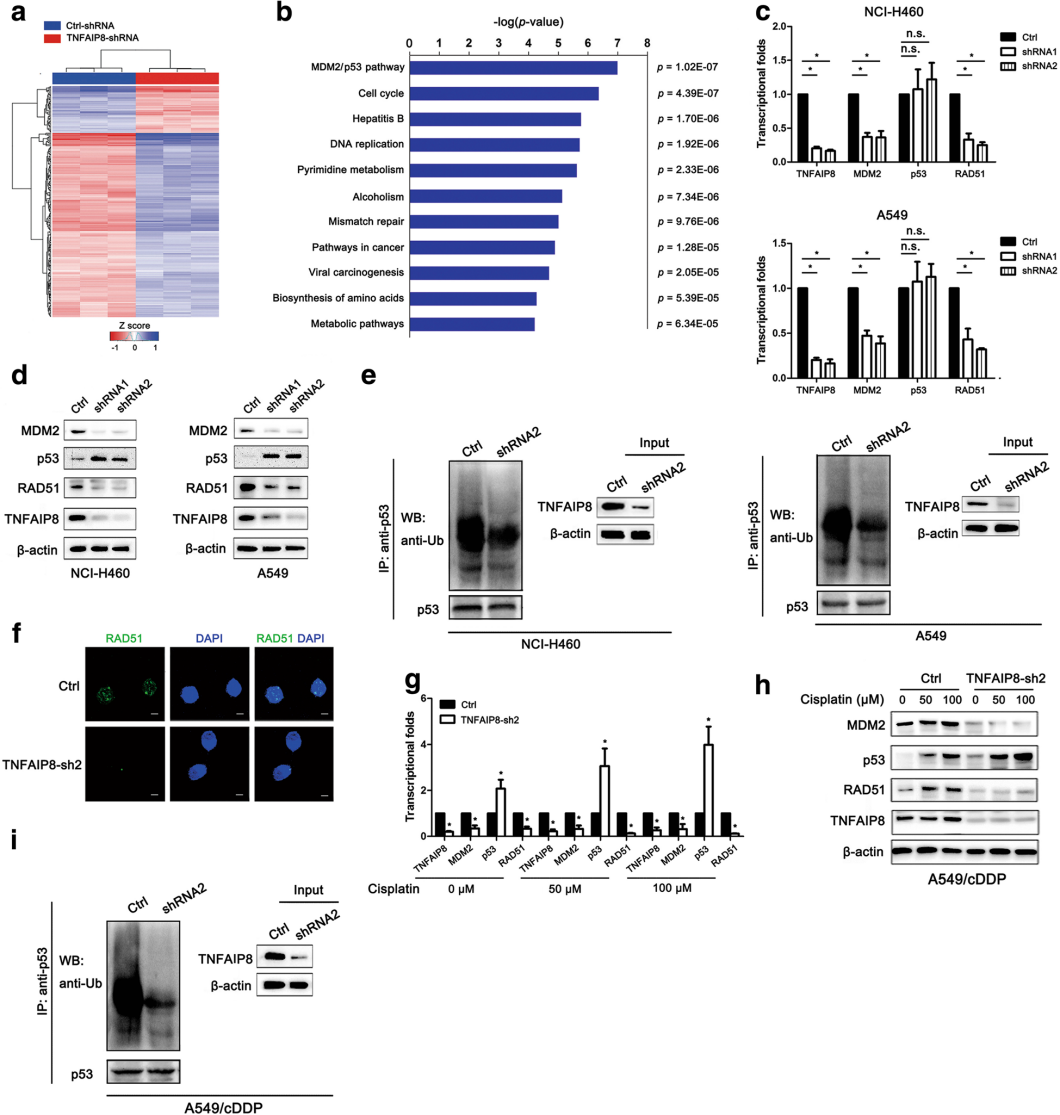
TNFAIP8 suppressed the p53 pathway in NSCLC cells. **a** NCI-H460 cells infected with lentivirus expressing either Ctrl-shRNA (blue) or TNFAIP8-shRNA2 (red). Genes and samples are listed in the rows and columns, respectively. A colour scale for the normalized expression data is shown at the bottom of the microarray heat map (blue represents downregulated genes; red represents upregulated genes). **b** Statistically significant modulation (indicated by the inverse log 10 of the *P* values) of Canonical Pathway following TNFAIP8 knockdown predicted by the commercially available IPA software. **c, d** qRT-PCR and western blot analyses of p53 and RAD51 expression levels in NCI-H460 and A549 cells treated with TNFAIP8 shRNAs. *P < 0.05 and n.s., not significant (Student’s t-test.) *P* values and n.s., not significant were calculated using Student’s t-test. **e** NCI-H460 and A549 cells infected with lentivirus encoding the indicated shRNA were treated with MG132 for 6 h. Lysates were immunoprecipitated with anti-p53 antibody. The ubiquitination of the p53 was analysed by western blotting using anti-ubiquitinantibody. **f** DNA repair after exposure to cisplatin was shown. A549/cDDP cells were transfected with control shRNA (Ctrl) or TNFAIP8 shRNA2 (TNFAIP8-sh2). Transfected cells were treated with 100 μM cisplatin for 48 h, and RAD51 foci were examined. Scale bar = 5 μM. **g, h** A549/cDDP cells were transfected with control shRNA (Ctrl) or TNFAIP8 shRNA2 (TNFAIP8-sh2) before cisplatin exposure. Cell mRNA and lysates were prepared after cisplatin exposure, and real-time qRT-PCR and western blotting analyses were performed. **i** A549/cDDP cells transfected with the indicated constructs were treated with MG132 for 6 h after cisplatin exposure. The ubiquitination of p53 was analysed as above. All n = 3; bar, SEM; n.s., no significant difference; *P < 0.05 (Student’s t-test)

TNFAIP8 is critical for tumour proliferation and chemoresistance in tumours. However, the mechanisms underlying TNFAIP8-mediated cancer development and its downstream pathways have not yet been systematically explored. Here, global gene expression profiling of NCI-H460 cells infected with lentivirus expressing either Ctrl-shRNA or TNFAIP8-shRNA2 was performed using a microarray (Fig. 5a). Using commercially available IPA software, we found that the MDM2/p53 pathway, cell cycle, hepatitis B, DNA replication, pyrimidine metabolism, alcoholism, and mismatch repair are among the top-ranked canonical pathways (Fig. 5b).

### TNFAIP8 regulates the MDM2/p53 pathway

As expected, TNFAIP8 silencing downregulated MDM2 expression and suppressed the expression levels of the DNA repair gene RAD51, as demonstrated through qRT-PCR and Western blot analyses (Fig. 5c, d). Notably, loss of TNFAIP8 did not affect the mRNA level of p53 but the protein level, suggesting that TNFAIP8 negatively regulates p53 stability at the post-translational level (Fig. 5c, d). Because MDM2 is the E3 ubiquitin ligase of p53 and binds to p53 to promote its degradation [19], we hypothesized that TNFAIP8 may be involved in the regulation of the ubiquitination of p53. As expected, when TNFAIP8 was knocked down, p53 ubiquitination was significantly decreased in NCI-H460 and A549 cells (Fig. 5e). RAD51, the expression of which is mediated by the negative regulation of p53, plays an important role in homologous recombination [24]. To assess whether TNFAIP8 impairs homologous recombination, we analysed the ability of A549/cDDP cells to form RAD51 foci in response to cisplatin. TNFAIP8 KD led to reduced RAD51 foci formation compared with the control 48 h after cisplatin treatment, confirming a defect in homologous recombination (Fig. 5f). Importantly, we found that “cisplatin” increased p53 levels and decreased the MDM2 and RAD51 levels in TNFAIP8 shRNA-treated cells compared with control shRNA-treated cells after cisplatin treatment (Fig. 5g, h). In addition, no significant changes in MDM2 protein expression were observed in cisplatin-treated A549/cDDP cells, but the protein levels of p53 were increased after cisplatin treatment, regardless of whether the cells were treated with TNFAIP8 shRNA 2 (Fig. 5h). As shown in Fig. 5i, knockdown of TNFAIP8 decreased p53 ubiquitination, when A549/cDDP cells were exposure to cisplatin. The above findings suggest that, after exposure to DNA damage stimuli, TNFAIP8 KD enhances DNA damage and decreases DNA repair.

### p53 is critical for the inhibition of NSCLC proliferation and cisplatin chemoresistance induced by TNFAIP8 KD

**Fig. 6:**
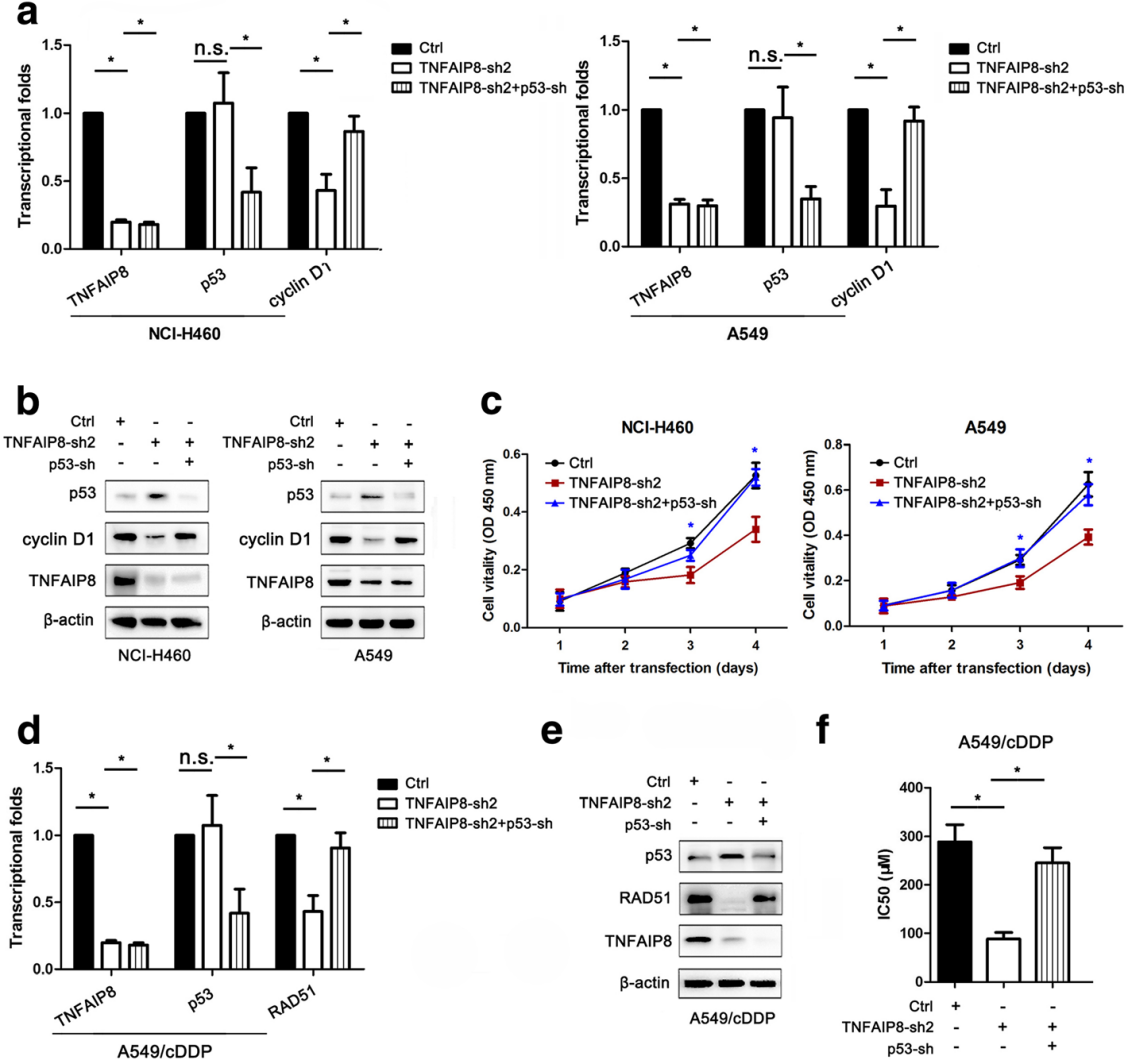
p53 inhibition is essential for the TNFAIP8 knockdown-mediated inhibition of NSCLC proliferation and cisplatin chemoresistance. **a**, **b** qRT-PCR and western blotting analyses showed that p53 shRNA (p53-sh) increased cyclin D1 expression levels in TNFAIP8 shRNA2 (TNFAIP8-sh2)-transfected NCI-H460 and A549 cells. *P < 0.05 and n.s., not significant (Student’s t-test.) **c** p53 inhibition restored NSCLC cell viability after TNFAIP8-sh2 transfection. **d**, **e** qRT-PCR and western blotting analyses showed that p53 shRNA (p53-sh) increased RAD51 expression levels in TNFAIP8 shRNA2 (TNFAIP8-sh2)-transfected A549/cDDP cells. *P < 0.05 and n.s., not significant (Student’s t-test.) **f** A549/cDDP cells were transfected with shRNA (Ctrl), TNFAIP8 shRNA2 (TNFAIP8-sh2) or p53 shRNA (p53-sh), and CCK-8 assay was used to measure cisplatin sensitivity. *P < 0.05 (Student’s t-test)

To determine whether the proliferation and cisplatin chemoresistance of lung cancer caused by TNFAIP8 resulted from the activation of p53 signalling, we transfected NCI-H460 and A549 cells with Ctrl-shRNA or TNFAIP8-shRNA2 and treated them (or not) with p53-shRNA (Fig. 6a, b).

As shown in Fig. 6c, TNFAIP8 KD-mediated suppression of NSCLC cell proliferation was inhibited by p53-shRNA. Similarly, qRT-PCR and Western blot analyses showed that the TNFAIP8 KD-mediated reductions in the cyclin D1 levels were also attenuated by p53-shRNA (Fig. 6a, b). Additionally, p53-shRNA reversed the increase in cisplatin chemosensitivity caused by TNFAIP8 KD in A549/cDDP cells (Fig. 6d-f). As expected, p53-shRNA also decreased the ability of TNFAIP8-shRNA2 to reduce the RAD51 expression levels (Fig. 6d, e).

### Effects of TNFAIP8 expression on the chemosensitivity of A549 cells to cisplatin in vivo

**Fig. 7:**
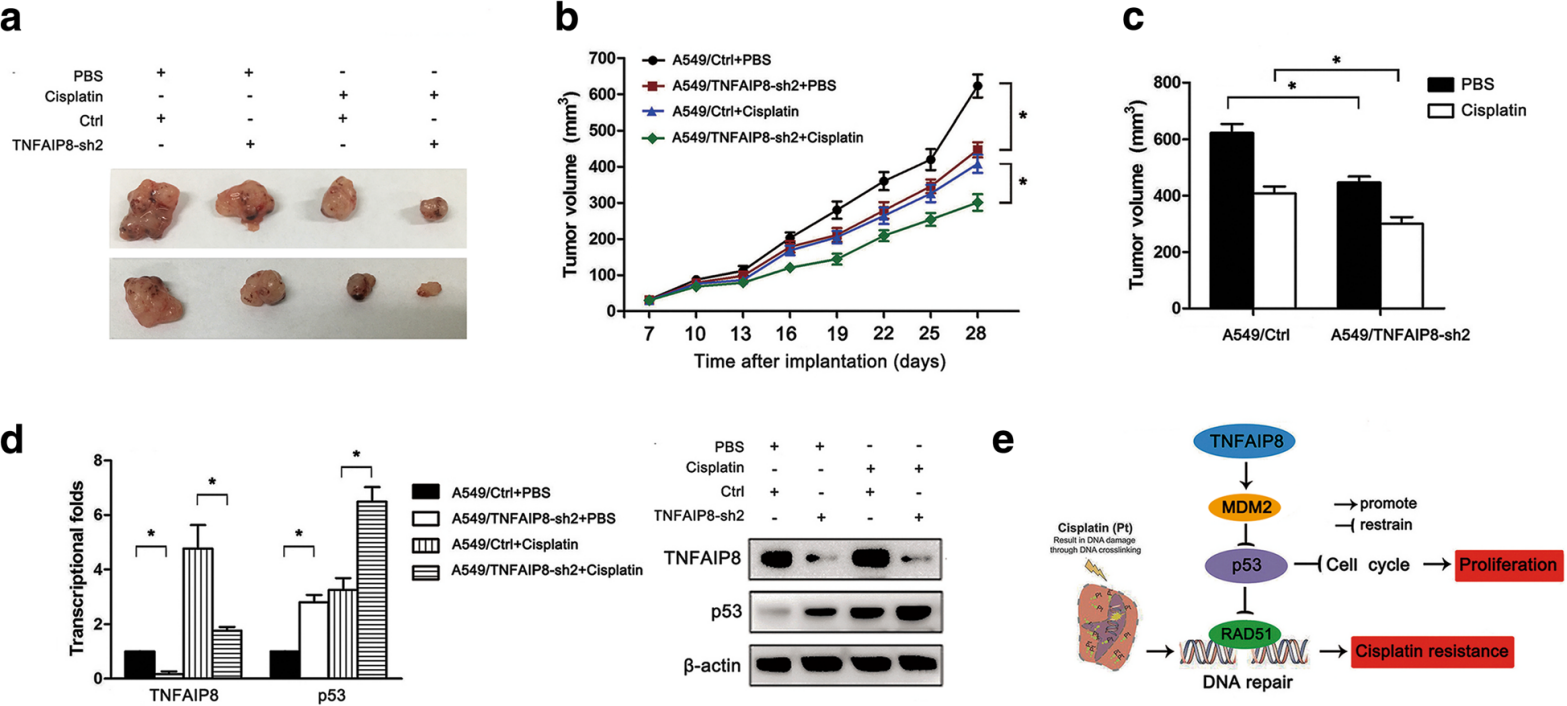
TNFAIP8 regulates NSCLC proliferation and chemoresistance in vivo. Mice were treated with cisplatin (3.0 mg/kg body weight; i.p., three times per week) or with 0.1 ml of PBS (pH 7.4; i.p., three times per week). **a** Representative features of tumours at 28 days after inoculation using A549/Ctrl or A549/TNFAIP8-sh2 cells treated with PBS or cisplatin. **b** Tumours growth in the mice injected with A549/Ctrl or A549/TNFAIP8-sh2 cells treated with PBS or cisplatin. Inoculations were performed in 10 mice. **c** Tumour volumes at day 28 after the inoculation. Left (black column), average tumour volumes at day 28 after inoculation with A549/Ctrl or A549/TNFAIP8-sh2 cells in mice treated with PBS; right (white column), average tumour volumes at day 28 after inoculation of A549/Ctrl or A549/TNFAIP8-sh2 cells in mice treated with cisplatin. **d** qRT-PCR and Western blot analysis of the relative p53 expression in tumours originating from A549/Ctrl or A549/TNFAIP8-sh2 cells treated with PBS or cisplatin. Data are presented as the mean ± SEM (n = 3). **e** Proposed model for TNFAIP8-induced proliferation and cisplatin resistance in NSCLC. TNFAIP8-mediated p53 suppression activates the expression of cyclin D1 and RAD51, thus promoting cell cycle progression and DNA repair. These actions lead to NSCLC cell proliferation and cisplatin resistance

We further investigated the role of TNFAIP8 on the in vivo sensitivity of NSCLC cells to cisplatin. NOD-SCID mice were randomly distributed and assigned to the following treatment groups: 1) A549/Ctrl + PBS, 2) A549/TNFAIP8-sh2 + PBS, 3) A549/Ctrl + cisplatin, or 4) A549/TNFAIP8-sh2 + cisplatin. The tumour volumes were measured every 3 days. Compared with the control shRNA group, the tumours in the A549/TNFAIP8-sh2 group exhibited statistically significant reduced tumour volumes (Fig. 7a-c). Although the combination of cisplatin and control shRNA led to decreased tumour volumes, the combination of A549/TNFAIP8-sh2 and cisplatin led to marked reductions in disease progression (Fig. 7a-c). The TNFAIP8 and p53 levels in tumour homogenates were then measured by qRT-PCR and Western blot analyses. We found that TNFAIP8 expression was significantly downregulated in xenografts formed from A549/TNFAIP8-sh2 cells and that p53 protein expression was significantly increased in these cells compared with A549/Ctrl cells (Fig. 7d). Additionally, the levels of p53 were increased after cisplatin treatment, and p53 levels were higher in the A549/TNFAIP8-sh2 + cisplatin group than in the A549/Ctrl + cisplatin group (Fig. 7d).

## Discussion

Our results revealed that TNFAIP8 expression was upregulated in tumour tissues from NSCLC patients and demonstrated that high TNFAIP8 expression is associated with advanced pT stage, advanced pTNM stage and lymph node metastasis. TNFAIP8 overexpression in NSCLC might also serve as a prognostic factor for poor OS and DFS. Similarly, high levels of TNFAIP8 expression in breast cancer, gastric cancer, pancreatic cancer, epithelial ovarian cancer and oesophageal squamous cell carcinoma correlates with tumour stage and lymph node metastasis and can predict poor survival [12–15]. These data suggest that TNFAIP8 can be considered a novel marker of lymph node metastasis and a promising therapeutic target for treating NSCLC patients.

Our biological results demonstrated that TNFAIP8 promoted NSCLC cell growth in vitro and in vivo by inducing G1-specific cyclin D1 and thus cell cycle transition from the G1 to the S phase. Consistent with our results, TNFAIP8 also plays a significant tumour-promoting role as an oncogene in various cancer types [5, 25–28]. Cyclins promote DNA synthesis by initiating cyclin-dependent kinase activation, retinoblastoma phosphorylation and E2F transcriptional factor activation [29]. Consistent with the role of TNFAIP8 in human tumour progression, Dong et al. demonstrated that TNFAIP8 inhibits Hippo transducer YAP phosphorylation, and this action leads to the upregulation of cyclin proteins and cell proliferation [27]. However, we demonstrated that the mechanism through which TNFAIP8 promotes NSCLC cell proliferation in vitro and tumour growth in vivo is dependent on p53. A previous study revealed that TNFAIP8 v2 supports DNA synthesis and might suppress the acetylation of p53 and the induction of p53 targets, particularly p21 [21]. However, there are no reports of the relationship between TNFAIP8 and wild-type p53.

MDM2 controls p53 levels by targeting it for ubiquitin-mediated proteasomal degradation in an auto-regulatory feedback loop, and the balance between MDM2 and ubiquitinated p53 would be disturbed if MDM2 protein levels are altered [19, 20] . Overexpression of MDM2 resulting in loss of p53 function confers resistance to DNA damage-inducing agents, such as cisplatin [20]. p53 as a nuclear phosphoprotein encoded is widely accepted as a critical tumour suppressor in cells [30]. p53 regulates downstream genes that are involved in cell cycle arrest, DNA repair and apoptosis [31]. p53 induces cell cycle arrest, primarily at the G1/S and G2/M checkpoints, by transactivating the downstream targets of p53, including CDKN1A, GADD45A, SFN and GTSE1 [32]. In the present study, we first found that TNFAIP8 could control the MDM2/p53 pathway in tumour cells. The detailed mechanisms of TNFAIP8-mediated MDM2 regulation have not been elucidated, and this work is ongoing in our laboratory.

The mechanism underlying cisplatin cytotoxicity is mediated by its ability to crosslink with the purine bases on DNA, and this action causes DNA damage and subsequently induces apoptosis in cancer cells [33]. Resistance to cisplatin-based chemotherapy is one of the major obstacles to treating NSCLC [34, 35]. We demonstrate that increased TNFAIP8 expression was associated with reduced postoperative survival, particularly in patients receiving adjuvant chemotherapy and advanced patients (Stage III). Our results agree with those of previous studies [12, 16], which indicate that TNFAIP8 is associated with platinum resistance in advanced cancer. This study provides the first demonstration that TNFAIP8 is involved in cisplatin resistance both in vitro and in vivo.

Increased DNA damage repair capacity has been reported as a hallmark mechanism of resistance to cisplatin [36]. RAD51, a highly conserved DNA repair protein, catalyzes DNA repair by forming nucleoprotein filaments and mediating strand exchange between DNA duplexes [37]. Thus, RAD51 has modulated cellular sensitivity to DNA-damaging treatments, such as cisplatin [38]. Our results indicate that RAD51 foci formation was decreased in TNFAIP8-deficient cells exposed to cisplatin. These results indicate that inhibiting TNFAIP8 might cause a defect in repairing DNA damage. In future mechanistic studies, the role of TNFAIP8 in regulation of RAD51 should be further explored.

Response to DNA damage, the wild-type p53 determines cell fate by facilitating DNA repair mechanisms [39]. However, p53 also inhibits inappropriate DNA repair by negatively regulating key homologous recombination proteins, such as RAD54 and RAD51 [40]. We found that TNFAIP8 negatively regulates p53 in NSCLC. These results explain why TNFAIP8 KD sensitizes NSCLC cells to cisplatin. Furthermore, our study suggested that TNFAIP8 induced RAD51 expression in a p53-dependent manner. Here, we propose a model in which the effects of TNFAIP8 on lung cancer progression and chemoresistance via targeting p53 and downregulating cyclin D1 and RAD51 (Fig. 7e).

## Conclusion

In conclusion, our study suggests that TNFAIP8 is a promising therapeutic target for treating NSCLC and highlights the value of using TNFAIP8 expression levels as a promising biomarker for NSCLC progression. Our study revealed that TNFAIP8 overexpression accelerates the G1/S phase transition via the MDM2/p53 pathway, inducing cell proliferation and ultimately leading to cisplatin chemoresistance. Simultaneously, the TNFAIP8-MDM2-p53 pathway regulates RAD51 and thus contributes to the response of NSCLC cells to cisplatin. Our characterization of this signalling pathway provides a better understanding of NSCLC development and progression and might provide novel therapeutic targets for the future treatment and reversal of cisplatin chemoresistance in NSCLC.

## Additional files

Additional file 1: **Figure S1.** Representative photomicrographs showing immune- histochemical staining for TNFAIP8. (a) Low expression in the ADC histotype. (b) High expression in the ADC histotype. (c) Low expression in the SCC histotype. (d) High expression in the SCC histotype. (Original magnification, × 400). ADC, Adenocarcinoma; SCC, Squamous cell carcinoma. (JPG 5442 kb)

Additional file 2: **Table S1.** Univariate and multivariate analyses of overall survival and disease-free survival. (DOC 56 kb)

## Abbreviations

A549/cDDP: Cis-diamminedichloroplatinum II resistant A549
DFS: Disease-free survival
IHC: Immunohistochemistry
IP: Immunoprecipitation
MDM2: Murine double minute 2
NSCLC: Non-small cell lung cancer
OS: Overall survival
qRT-PCR: quantitative Real-time reverse transcription-polymerase chain reaction
TNFAIP8: Tumour necrosis factor alpha-induced protein 8
