# Supplementary figures and images for: TNFAIP8 promotes the proliferation and cisplatin chemoresistance of non-small cell lung cancer through MDM2/p53 pathway
Source: Cell Commun Signal. 2018 Jul 31;16:43. doi: 10.1186/s12964-018-0254-x (PMC6069800; doi:10.1186/s12964-018-0254-x)

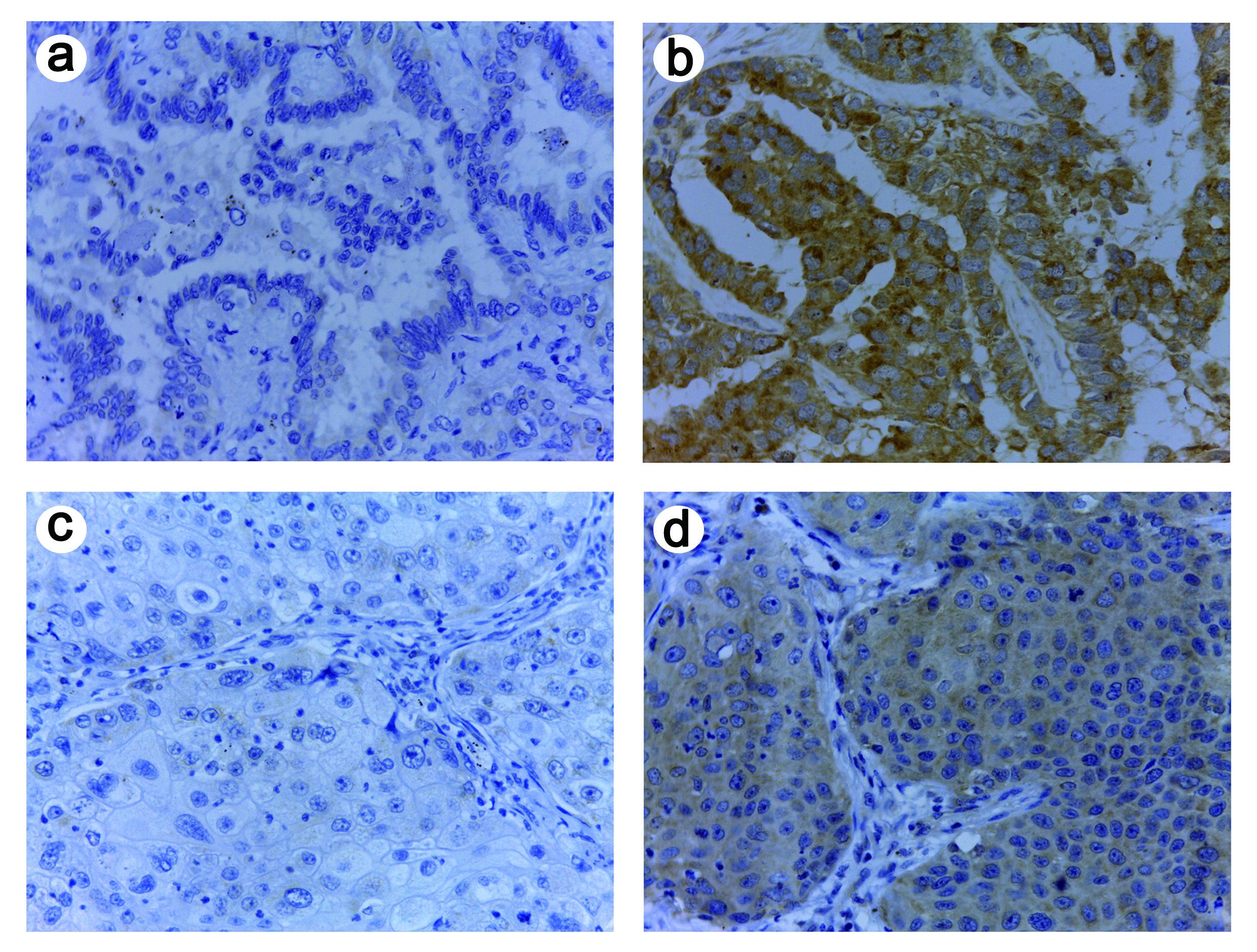

Supplement: Supplementary file 1 — Figure S1. Representative photomicrographs showing immune- histochemical staining for TNFAIP8. (a) Low expression in the ADC histotype. (b) High expression in the ADC histotype. (c) Low expression in the SCC histotype. (d) High expression in the SCC histotype. (Original magnification, × 400). ADC, Adenocarcinoma; SCC, Squamous cell carcinoma. (JPG 5442 kb) [file 12964_2018_254_MOESM1_ESM.jpg]
