# Supplementary material for: TNFAIP8 promotes the proliferation and cisplatin chemoresistance of non-small cell lung cancer through MDM2/p53 pathway
Source: Cell Commun Signal. 2018 Jul 31;16:43. doi: 10.1186/s12964-018-0254-x (PMC6069800; doi:10.1186/s12964-018-0254-x)
Supplement: Supplementary file 2 — Table S1. Univariate and multivariate analyses of overall survival and disease-free survival. (DOC 56 kb) [file 12964_2018_254_MOESM2_ESM.doc]

Additional file 2: Table S1 Univariate and multivariate analyses of overall survival and disease-free survival.

|  | OS | | | DFS | | |
| --- | --- | --- | --- | --- | --- | --- |
|  | Univariate analysis | Multivariate analysis | | Univariate analysis | Multivariate analysis | |
| Variable | *P* | HR (95% CI) | *P* | *P* | HR (95% CI) | *P* |
|  |  |  |  |  |  |  |
| Age |  |  |  |  |  |  |
| <60 |  |  |  |  |  |  |
| ≥60 | 0.932 | － | － | 0.705 | － | － |
|  |  |  |  |  |  |  |
| Gender |  |  |  |  |  |  |
| Female |  |  |  |  |  |  |
| Male | 0.373 | － | － | 0.759 | － | － |
|  |  |  |  |  |  |  |
| Differentiation |  |  |  |  |  |  |
| Good |  |  |  |  |  |  |
| Moderate |  |  |  |  |  |  |
| Poor | 0.975 | － | － | 0.941 | － | － |
|  |  |  |  |  |  |  |
| Histological cell type |  |  |  |  |  |  |
| Squamous cell carcinoma |  |  |  |  |  |  |
| Adenocarcinoma | 0.246 | － | － | 0.036* | 1.317 (0.836 to 2.077) | 0.235 |
|  |  |  |  |  |  |  |
| pT stage |  |  |  |  |  |  |
| I |  |  |  |  |  |  |
| II |  |  |  |  |  |  |
| III | 0.672 | － | － | 0.566 | － | － |
|  |  |  |  |  |  |  |
| pTNM stage |  |  |  |  |  |  |
| I |  |  |  |  |  |  |
| II |  |  |  |  |  |  |
| III | <0.001* | 1.511 (0.883 to 2.586) | 0.132 | <0.001* | 1.502 (0.940 to 2.400) | 0.089 |
|  |  |  |  |  |  |  |
| Lymph node metastasis |  |  |  |  |  |  |
| Present |  |  |  |  |  |  |
| Absent | <0.001* | 1.554 (0.601 to 4.017) | 0.363 | 0.001* | 1.060(0.467 to 2.404) | 0.890 |
|  |  |  |  |  |  |  |
| TNFAIP8 expression |  |  |  |  |  |  |
| Low |  |  |  |  |  |  |
| High | 0.015* | 1.858 (1.164 to 2.966) | 0.009* | 0.046* | 1.605 (1.056 to 2.440) | 0.027* |

Abbreviations: NSCLC = non-small cell lung cancer; pTNM stage = Tumor, node, metastasis (pathological stage); p T = pathological T stage; n = number of patients; OS = overall survival; DFS = disease-free survival; HR = hazard ratio; CI = confidence interval. pTNM stage, Lymph node metastasis and TNFAIP8 expression were selected for multivariate analysis for OS. Histological cell type, pTNM stage, Lymph node metastasis and TNFAIP8 expression were selected for multivariate analysis for DFS. **P*< 0.05 was considered statistically significant.
